# First clinical experience with the surpass elite flow diverter in the treatment of intracranial aneurysms

**DOI:** 10.1007/s00234-026-04010-y

**Published:** 2026-05-02

**Authors:** Om H. Gandhi, Sami Almasri, Warda Ahmed, Suraj R. Dumasia, Nathan Yu, Mohammad S. Rashad, Linda J. Bagley, Matthew Fiesta, Omar A. Choudhri

**Affiliations:** 1 Department of Neurosurgery, University of Pennsylvania, Philadelphia, United States; 2 Aga Khan University, Karachi, Pakistan; 3 Department of Neurosurgery, University of Pennsylvania, Philadelphia, United States; 4 Medical School, University of Michigan–Ann Arbor, Ann Arbor, United States; 5 Department of Radiology, University of Pennsylvania, Philadelphia, United States; 6 Radiology Associates of North Texas, Fort Worth, United States

**Keywords:** Surpass elite, Flow diverter, Intracranial aneurysm, Aneurysm occlusion, Neurointervention

## Abstract

**Background:**

The Surpass Elite (Stryker Neurovascular) is a third-generation flow diverter featuring modified braid architecture with increased braid angle and novel surface charge neutralization treatment. This study presents the first clinical experience with the Surpass Elite globally.

**Methods:**

A retrospective analysis of 81 consecutive cases (78 unique patients) treated with the Surpass Elite across four centers by two operators from January to July 2025 was performed. Data collected included patient demographics, aneurysm characteristics, procedural details, technical success, complications, and angiographic/clinical outcomes. Primary endpoints were technical success rate, perioperative safety profile, and aneurysm occlusion rates at approximately 6-month follow-up.

**Results:**

The cohort comprised 78 unique patients (76.9% female, mean age 59.2 ± 14.5 years) with 81 aneurysms treated using 112 total devices (mean 1.38 devices per case). Aneurysms were predominantly located in the internal carotid artery (75.3%). Technical success was achieved in 100% of cases regardless of access site. Balloon angioplasty was required in only 13.6% of cases. Among 61 aneurysms with complete angiographic follow-up at a mean of 6 months, complete occlusion (RROC Class I or OKM Grade D) was achieved in 78.7%. In-stent stenosis (< 50%) was observed in only 3.3% of cases with follow-up imaging, suggesting favorable vessel remodeling with minimal intimal hyperplasia. The immediate intraprocedural complication rate was 2.5% (2/81), including one device-related microperforation (complete recovery) and one intraoperative thrombosis (managed successfully intraprocedurally; delayed stroke from subsequent medication noncompliance). Delayed complications within 30 days included one stroke from antiplatelet noncompliance (fatal) and one access-site hematoma (recovered). One delayed adverse event beyond 30 days (delayed right temporal intraparenchymal hemorrhage at 10 months) was observed in a patient on dual antiplatelet therapy plus apixaban and was attributed to cerebral amyloid angiopathy; this event was not considered device- or procedure-related. Overall mortality was 7.4% (6/81): one death (1.2%) was procedure-related (stroke from medication noncompliance), one death occurred within 30 days from post-SAH vasospasm in a ruptured aneurysm case, and four deaths occurred after 30 days (recurrent ICH at 3 weeks, acute respiratory failure with acute on chronic heart failure at 5 months, homicide at approximately 6 months, and one of unknown cause), none of which were device- or procedure-related.

**Conclusion:**

This first published clinical experience with Surpass Elite flow diverter demonstrates reliable technical deliverability, favorable early occlusion rates comparable to established flow diverters, and a satisfactory safety profile. The low rates of required balloon angioplasty support the clinical benefit of the modified braid architecture. These preliminary findings support the continued clinical evaluation of the Surpass Elite in larger prospective cohorts.

**Supplementary Information:**

The online version contains supplementary material available at 10.1007/s00234-026-04010-y.

## Introduction

Flow diversion has improved the endovascular treatment of intracranial aneurysms, particularly for large, wide-necked, and complex lesions difficult to treat with conventional coiling or stent-assisted techniques [1–3]. By redirecting blood flow from the aneurysm sac flow diverters (FDs) promote progressive thrombosis and eventual aneurysm obliteration [4, 5]. Since the introduction of the Pipeline Embolization Device (PED) in 2011, multiple FDs emerged incorporating design modifications improving deliverability, apposition, and clinical outcomes [5, 6].

The Surpass FD system (Stryker Neurovascular, Fremont, California) has undergone iterative design evolution since its initial release. The first-generation Surpass device received CE mark approval in 2011 but faced challenges with deliverability, vessel conformability, and lacked radiopaque markers for fluoroscopic visibility [7–9]. The second-generation Surpass Evolve, introduced in 2019, offered improvements including enhanced flexibility and adaptability with maintained radial force, leading to successful technical performance and FDA approval following the SCENT trial [10]. However, a meta-analysis of nine studies revealed the Evolve required balloon angioplasty to enhance wall apposition in 26.1% of reported cases [11]. Surpass Elite is the third-generation FD with a modified braid for better spontaneous opening, enhanced heat treatment for smoother deployment, and a novel biopassive surface on its 64-wire cobalt-chromium construction to reduce thrombogenicity while maintaining integrity [12, 13].

Despite FDA approval in June 2024 and market release, clinical outcome data for Surpass Elite remains absent from the peer-reviewed literature. This study presents the first published clinical experience with the Surpass Elite, reporting technical success, safety, and early angiographic outcomes from an 81-case, four-center, two-physician cohort. The study aims to provide preliminary evidence for Surpass Elite performance and provide practical insights for clinicians considering integration of this device into practice.

## Methods

### Study design and patient selection

A retrospective analysis was performed of consecutive patients who underwent intracranial aneurysm treatment with the Surpass Elite FD at four medical centers between January 2025 and July 2025. All procedures were performed by two experienced neurointerventionalists with FD experience. Institutional Review Board approval was obtained at participating institutions, with consent waived given the retrospective nature of the analysis. Inclusion criteria encompassed all patients treated with the Surpass Elite device, with no exclusion criteria applied so as to ensure representation of real-world clinical practice.

### Device specifications

The Surpass Elite (Stryker Neurovascular, Fremont, California) is a 64-wire cobalt-chromium flow diverter delivered through a 0.027-inch inner diameter microcatheter (Excelsior XT-27). The implant consists of 52 structural cobalt-chromium wires and 12 platinum-tungsten wires for fluoroscopic visibility, available in eight diameters (3.25–5.25 mm) and lengths from 12 to 50 mm (Supplementary Table S1). Compared to its predecessor, the Surpass Evolve (four diameters, 3.25–5.0 mm; lengths 12–40 mm), the Elite offers a broader size matrix with the addition of a 5.25 mm diameter and 50 mm length option [13, 14].

**Fig. 1:**
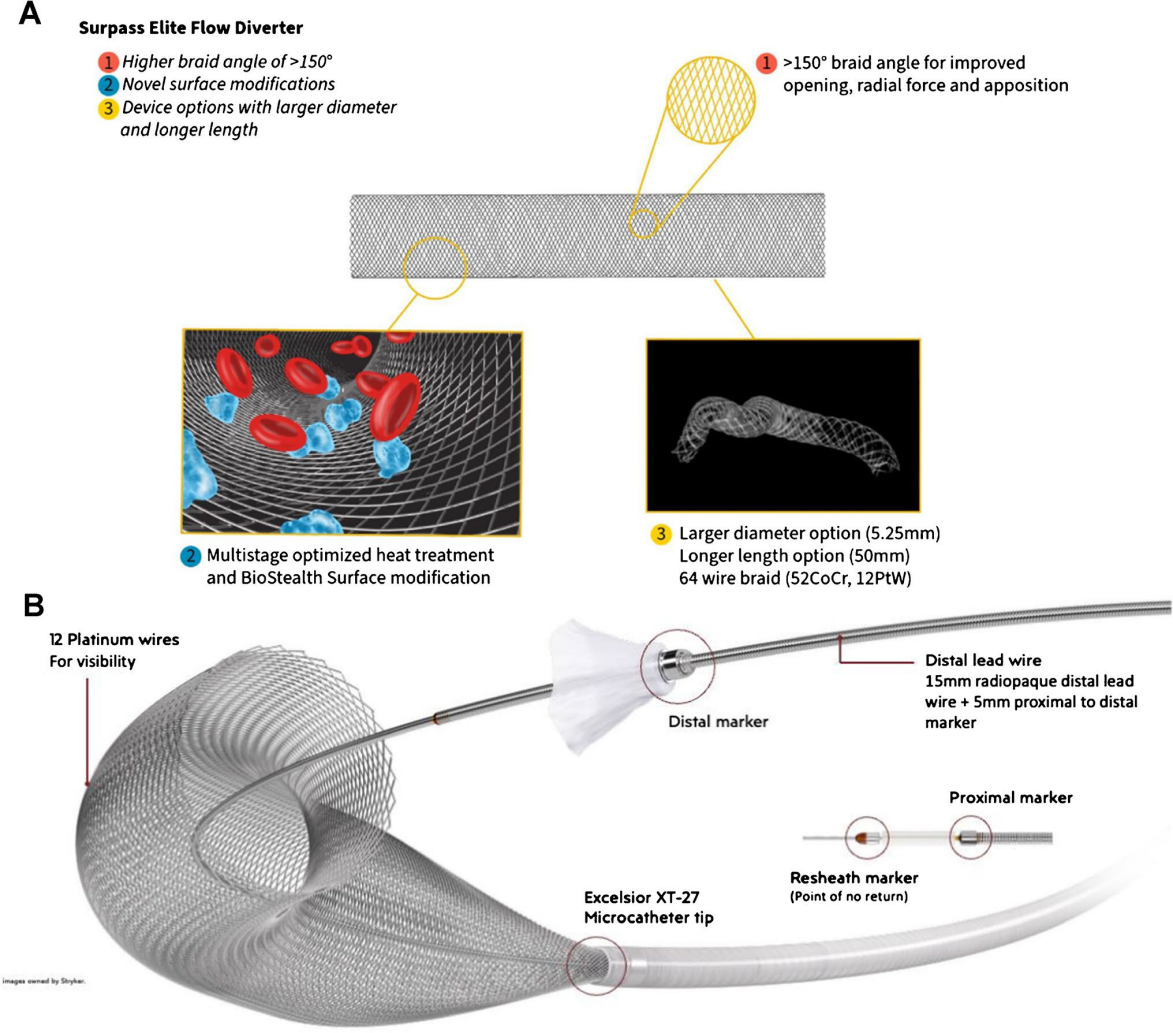
Key features and deployment mechanism of the Surpass Elite flow diverter. **A** The Surpass Elite features a > 150° braid angle for improved opening, radial force, and apposition. The device consists of a 64-wire braid (52 cobalt-chromium alloy wires and 12 platinum wires for radiopacity), shown here on 3D rotational angiography with two overlapping devices. Novel surface modifications include multistage optimized heat treatment and BioStealth surface modification with neutral surface charge to reduce thrombogenicity. The device offers expanded sizing options with a larger diameter (5.25 mm versus 5.0 mm for Evolve) and longer length (50 mm versus 40 mm for Evolve). **B** Device structure and deployment mechanism showing distal lead wire (15 mm radiopaque distal lead wire + 5 mm proximal to distal marker), distal marker, proximal marker, resheath marker (point of no return), and Excelsior XT-27 microcatheter tip with 12 platinum wires for visibility. (Illustration courtesy of Stryker Neurovascular)

Key design modifications include an increased braid angle (150–158° vs. 133–152° for the Evolve), which promotes improved spontaneous device opening and wall apposition, and a multistage optimized heat treatment that enhances material strength and deployment responsiveness [13]. The greater foreshortening range (42–61% vs. 35.5–59%) must be accounted for during sizing. The delivery system features a petal mechanism, anatomy-specific solid core delivery wire, and upgraded resheath pad, consistent with the Evolve platform **(**Fig. 1**)** [13–15].

The BioStealth surface modification alters the outermost 4 nm of the cobalt-chromium surface to equalize the distribution of positively and negatively charged surface compounds, producing a more neutrally charged and less reactive surface [13]. This limits conformational activation of adsorbed plasma proteins—particularly fibrinogen and Factor XII—thereby attenuating intrinsic coagulation cascade activation and thrombin generation [16, 17]. In vitro testing demonstrated a 91% reduction in peak thrombin generation compared to an unmodified device, and a GLP endothelialization study in 18 rabbits showed comparable healthy neointima at 30 days. This approach differs from the phosphorylcholine coating on the Pipeline Shield, which mimics the erythrocyte membrane to resist platelet adhesion [18–20]. Detailed bench and preclinical performance data are presented in Supplementary Table S2. The Surpass Elite received FDA clearance in June 2024 and CE mark approval in September 2024.

### Data collection

Data were extracted from electronic medical records, procedural reports, and imaging studies. Collected variables included patient demographics (age, sex, race/ethnicity, comorbidities, smoking history, family history of aneurysms), aneurysm characteristics (location, dimensions, morphology, configuration, rupture status, prior treatment), parent vessel diameters (proximal and distal), procedural details (access site, antiplatelet regimen, P2Y12 assay values, number and size of devices, adjunctive techniques, anesthesia type, procedure duration, fluoroscopy time, radiation dose, contrast volume, postoperative imaging), technical outcomes (device deployment success, apposition), complications (intraoperative and postoperative events with detailed characterization), and follow-up data (angiographic occlusion status using Raymond-Roy Occlusion Classification [RROC] and O’Kelly-Marotta [OKM] grading scales, in-stent stenosis, follow-up imaging modality, clinical functional status using modified Rankin Scale [mRS], antiplatelet regimen, mortality) [21, 22].

### Procedural technique

All patients received dual antiplatelet therapy (DAPT) a week prior to elective procedures, typically consisting of aspirin 81–325 mg daily and either Plavix (clopidogrel) 75 mg, Brilinta (ticagrelor) 90 mg twice daily, or Effient (prasugrel) 10 mg daily. Platelet function testing was performed when available; at centers without platelet function testing capability, patients were started on aspirin and clopidogrel and monitored for thrombus formation after device deployment, with Integrilin administration and conversion to ticagrelor if thrombus was observed. For ruptured aneurysms, antiplatelet therapy was initiated as soon as clinically feasible, often with loading doses administered during or immediately following the procedure. In cases involving large or giant aneurysms, perioperative dexamethasone was administered (4 mg twice daily, tapered over 72 h) to mitigate potential thrombosis-induced perianeurysmal inflammation [23–25].

Procedures were largely performed under general anesthesia with full systemic heparinization targeting an activated clotting time of 250–300 s. Vascular access was obtained via transradial, transfemoral, or transulnar approach based on operator preference and patient anatomy. Both transradial and transfemoral access routes were utilized when initial approach failed. Standard neurointerventional technique was employed for guide catheter positioning and microcatheter navigation. All cases employed a tri-axial catheter setup with the most common guide catheters used being Ballast (Balt USA, Irvine, California), Zebra (Q’Apel Medical, Fremont, California), and RIST (Medtronic, Dublin, Ireland). The intermediate catheters employed were either Vecta 46 (Stryker Neurovascular, Fremont, California), AXS Apro 55 (Alembic, Mountain View, California), or Catalyst 5 (Stryker Neurovascular, Fremont, California). The microcatheter used was XT-27 (Stryker Neurovascular, Fremont, California) and microwires employed were a mix of Synchro Select (Stryker Neurovascular, Fremont, California), Aristotle 14 or 24 (Scientia Vascular, West Valley City, Utah), or Drivewire 24 (Rapid Medical, Yokneam, Israel). The Surpass Elite (Stryker Neurovascular, Fremont, California) devices were selected based on parent vessel diameter measurements from pre-procedural angiography, typically sizing the device 0.5–1.0 mm larger than the proximal vessel diameter to ensure adequate wall apposition and based on sizing software (Angiosuite, Oculus Imaging, Knoxville, Tennessee).

Device deployment followed manufacturer recommendations [13]. Following deployment, repeat angiography was performed to assess device apposition, parent vessel patency, and aneurysm filling. Balloon angioplasty was performed at operator discretion when incomplete device opening was observed. Adjunctive coil embolization was performed in select cases to promote earlier thrombosis in large or giant aneurysms or when significant intra-procedural rupture risk was present.

Postoperatively, patients typically underwent cone-beam CT with 20% contrast dilution in the angiography suite to assess for device apposition, braid architecture and any acute complications. Patients were maintained on DAPT for a minimum of 6 months following the procedure, with subsequent transition to aspirin monotherapy in the setting of aneurysm obliteration.

### Follow-up protocol & outcome measures

Clinical follow-up included neurological examination at hospital discharge and subsequent outpatient visits at approximately 1, 3, and 6 months. Angiographic follow-up was typically performed at 6 months using digital subtraction angiography or, when clinically appropriate, non-invasive imaging with CT or angiography.

Technical success was defined as successful deployment of the Surpass Elite device(s) in the intended location with restoration of antegrade flow. Complete aneurysm occlusion was defined as RROC Class I or OKM Grade D. Adequate aneurysm occlusion was defined as RROC Class I-II or OKM Grade C-D per established literature definitions [21, 22]. Good functional outcome was defined as a modified Rankin Scale score (mRS) of 0–2. Procedural complications included any adverse event occurring during or within 30 days of the procedure. Procedure-related mortality was defined as death directly attributable to the flow diversion procedure or its immediate complications. In-stent stenosis was assessed on follow-up imaging. Retreatment was defined as any prior intervention performed on the targeted aneurysm. Aneurysms were classified per their maximum dimension, consistent with standard definitions in the literature.

### Statistical analysis

Descriptive statistics were calculated for all variables. Categorical variables are presented as frequencies and percentages. Continuous variables were assessed for normality using the Shapiro–Wilk test with those demonstrating normal distribution reported as mean ± standard deviation, while non-normally distributed variables were reported as median (interquartile range [IQR]). For subgroup analyses comparing outcomes across different patient and procedural characteristics, Fisher’s exact test was used given the relatively small sample sizes and sparse contingency tables. A two-sided *p*-value < 0.05 was considered statistically significant. All statistical analyses were performed using RStudio (version 2024.12.0) with R version 4.5.2 (R Foundation for Statistical Computing, Vienna, Austria). A minimum follow-up interval of 3 months was required for an aneurysm to be included in the formal angiographic occlusion analysis (RROC and OKM scoring), given the progressive nature of flow diversion–induced thrombosis; cases with imaging obtained at shorter intervals were captured clinically but excluded from the occlusion denominators.

## Results

### Patient demographics and baseline characteristics

**Fig. 2:**
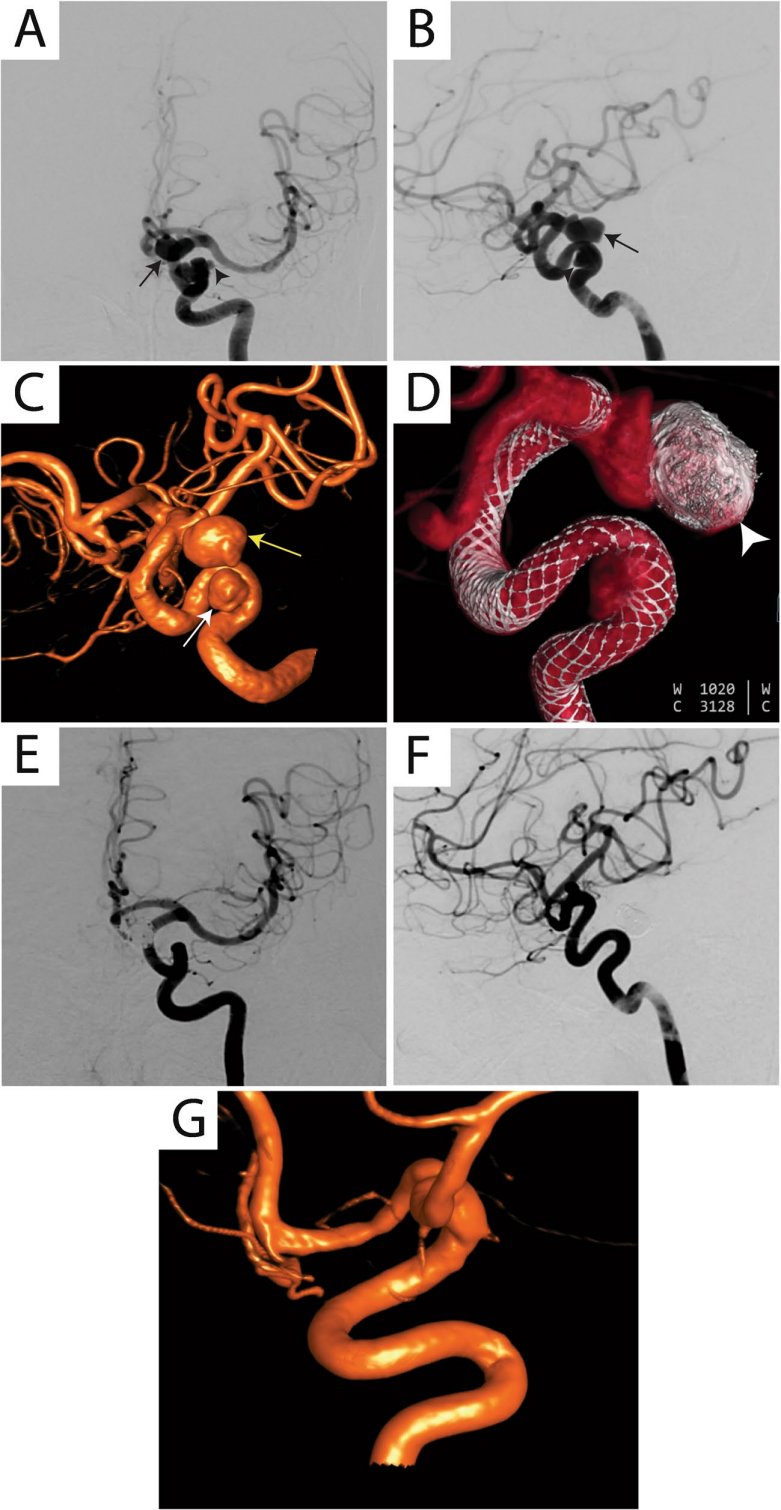
69-year-old Black male with multiple unruptured left internal carotid artery (ICA) aneurysms who presented with multiple intracranial aneurysms and left ear hearing issues. **A**, **B** Anteroposterior (AP) and lateral views of left ICA angiographic injection, demonstrating a large left posterior communicating artery aneurysm measuring 12.0 × 8.5 × 10.3 mm with a 5.3 mm neck (black arrow) and a left cavernous ICA aneurysm measuring 7.5 × 7.4 × 5.8 mm with a 5.1 mm neck (black arrowhead). **C** Pretreatment rotational 3D angiogram of left ICA showing the left posterior communicating artery aneurysm (yellow arrow) and left cavernous ICA aneurysm (white arrow). **D** Immediate post-treatment dual-volume rotational 3D angiogram of left ICA showing two overlapping Surpass Elite devices (4.5 × 25 mm and 5.0 × 40 mm) with coil mass inside the larger aneurysm (white arrowhead). **E**, **F** AP and lateral views of left ICA angiographic injection at 6-month follow-up post-treatment, demonstrating complete obliteration of both aneurysms. **G** Rotational 3D angiogram of left ICA at 6-month follow-up showing no residual aneurysm filling and smooth native vessel remodeling

A total of 81 aneurysms in 78 unique patients were treated across 80 procedures. Most procedures treated a single aneurysm; one procedure in one patient treated two aneurysms simultaneously with overlapping flow diverter constructs (illustrated in Fig. 2). Two additional patients underwent two separate procedures for two distinct aneurysms at different time points (Table 1). The cohort was predominantly female (76.9%, *n* = 60) with a median age of 62 years (range: 21–84 years). The racial/ethnic distribution included White/Caucasian (48.7%, *n* = 38), Black (17.9%, *n* = 14), Hispanic (17.9%, *n* = 14), Asian (6.4%, *n* = 5), and Other/Unknown (9.0%, *n* = 7). Comorbidities were common, with hypertension and hyperlipidemia present in 61.5% and 60.3% of patients, respectively. A family history of aneurysms was documented in 15.4% (*n* = 12) of patients.

**Table 1:** Patient demographics (*N* = 78)

Variable	Total (*N* = 78)
Age, years	
Mean ± SD	59.2 ± 14.5
Median	62
Range	21–84
Sex	
Female	60 (76.9%)
Male	18 (23.1%)
Race/Ethnicity	
White/Caucasian	38 (48.7%)
Black	14 (17.9%)
Hispanic	14 (17.9%)
Asian	5 (6.4%)
Other/Unknown	7 (9.0%)
Comorbidities	
Hypertension	48 (61.5%)
Hyperlipidemia	47 (60.3%)
Smoking History	
Never	34 (43.6%)
Former	34 (43.6%)
Current	9 (11.5%)
Unknown	1 (1.3%)
Family History of Aneurysms	12 (15.4%)

Values are n (%) unless otherwise specified

### Aneurysm characteristics

Of the 81 treated aneurysms (Table 2), the majority presented incidentally (28.4%, *n* = 23), with headache (21.0%, *n* = 17), stroke/TIA (11.1%, *n* = 9), dizziness/vertigo (7.4%, *n* = 6), and subarachnoid hemorrhage (SAH) (6.2%, *n* = 5) representing other most common presentations.

**Table 2:** Aneurysm characteristics (*N* = 81 cases)

Variable	Total (*N* = 81 cases)
Clinical Presentation	
Incidental	23 (28.4%)
Headache	17 (21.0%)
Stroke/TIA	9 (11.1%)
Dizziness/Vertigo	6 (7.4%)
SAH	5 (6.2%)
Visual symptoms	3 (3.7%)
Cranial nerve deficit	2 (2.5%)
Pulsatile tinnitus	2 (2.5%)
Other	14 (17.3%)
Location	
Internal Carotid Artery	61 (75.3%)
Posterior Circulation	14 (17.3%)
Middle Cerebral Artery	2 (2.5%)
ACA/AComA	2 (2.5%)
Other	2 (2.5%)
Aneurysm Size	
Maximum dimension, mm, mean ± SD	6.2 ± 5.4
Neck width, mm, mean ± SD (*n* = 73)	3.5 ± 2.5
Size Category	
< 3 mm	13 (16.0%)
3–5 mm	33 (40.7%)
5–7 mm	18 (22.2%)
7–12 mm	13 (16.0%)
13–25 mm	1 (1.2%)
> 25 mm (Giant)	3 (3.7%)
Morphology	
Saccular	69 (85.2%)
Fusiform	9 (11.1%)
Fusiform with saccular component	1 (1.2%)
Blister	1 (1.2%)
Pseudoaneurysm	1 (1.2%)
Configuration	
Sidewall	67 (82.7%)
Fusiform	10 (12.3%)
Bifurcation	4 (4.9%)
Rupture Status	
Unruptured	77 (95.1%)
Ruptured	4 (4.9%)
Number of Aneurysms	
Single	70 (86.4%)
Multiple	11 (13.6%)
Prior Treatment	
De novo (no prior treatment)	71 (87.7%)
Previously treated	9 (11.1%)
Prior flow diverter (FRED X)	3
Prior flow diverter (Surpass Evolve)	2
Prior flow diverter (Pipeline)	1
Prior coil embolization	3
Parent Vessel Diameter, mm (*n* = 77)	
Proximal, mean ± SD	3.91 ± 0.82
Distal, mean ± SD	3.24 ± 0.81

Values are n (%) unless otherwise specified

Most aneurysms were in the internal carotid artery (ICA) (75.3%, *n* = 61), followed by the posterior circulation (17.3%, *n* = 14), middle cerebral artery (2.5%, *n* = 2), and ACA/AComA (2.5%, *n* = 2). The mean maximum aneurysm diameter was 6.2 ± 5.4 mm, and mean neck width was 3.5 ± 2.5 mm (*n* = 73 with measurements). By size category, 16.0% (*n* = 13) were < 3 mm, 40.7% (*n* = 33) were 3–5 mm, 22.2% (*n* = 18) were 5–7 mm, 16.0% (*n* = 13) were 7–12 mm, 1.2% (*n* = 1) were 13–25 mm, and 3.7% (*n* = 3) were giant (> 25 mm).

Most aneurysms were saccular in morphology and sidewall in configuration (85.2% and 82.7%, respectively), with fusiform presentation in 11.1% (*n* = 9). 95.1% (*n* = 77) of aneurysms were unruptured with 13.6% (*n* = 11) of patients having multiple aneurysms. Prior treatment of the target aneurysm was documented in 11.1% (*n* = 9) of cases. Mean parent vessel diameters were 3.91 ± 0.82 mm proximally and 3.24 ± 0.81 mm distally (*n* = 77 cases with measurements).

### Procedural characteristics

Preoperative imaging modalities included digital subtraction angiography (DSA) in 63.0% (*n* = 51), computed tomography angiography (CTA) in 21.0% (*n* = 17), magnetic resonance angiography/imaging (MRA/MRI) in 14.8% (*n* = 12), and computed tomography (CT) in 1.2% (*n* = 1) of cases (Table 3). Dual antiplatelet regimens, combining aspirin with Plavix (63.0%, *n* = 51), Brilinta (33.3%, *n* = 27), Effient (1.2%, *n* = 1), apixaban (1.2%, *n* = 1), or warfarin (1.2%, *n* = 1), were used.

**Table 3:** Procedural characteristics (*N* = 81 cases)

Variable	Total (*N* = 81 cases)
Preoperative Imaging (prior to day of treatment)	
DSA	51 (63.0%)
CTA	17 (21.0%)
MRA/MRI	12 (14.8%)
CT head without contrast	1 (1.2%)
Antiplatelet Regimen	
ASA + Plavix	51 (63.0%)
ASA + Brilinta	27 (33.3%)
ASA + Effient	1 (1.2%)
ASA + Apixaban	1 (1.2%)
ASA + Warfarin	1 (1.2%)
P2Y12 value, mean ± SD (*n* = 59)	102.2 ± 80.7
Access Site	
Radial	43 (53.1%)
Femoral	34 (42.0%)
Ulnar	2 (2.5%)
Both (radial + femoral)	2 (2.5%)
Number of Surpass Elite Devices	
1 device	61 (75.3%)
2 devices	17 (21.0%)
≥ 3 devices	3 (3.7%)
Mean devices per case	1.38
Total devices deployed	112
Adjunctive Techniques	
Balloon Angioplasty	11 (13.6%)
Coil Embolization	8 (9.9%)
Procedure Metrics	
Procedure duration, min (*n* = 42)	
Mean ± SD	84.4 ± 47.0
Median (IQR)	72 (60–84)
Fluoroscopy time, min (*n* = 81)	
Mean ± SD	37.0 ± 23.9
Median (IQR)	31.8 (25.5–40.8)
Radiation dose, mGy·cm (*n* = 80)	
Median (IQR)	1750.8 (1016.7–2801.5)
Contrast volume, mL (*n* = 46)	
Mean ± SD	81.5 ± 32.5
Median (IQR)	75 (50–100)
Anesthesia: General (*n* = 47)	46/47 (97.9%)
Postoperative Dyna CT	40 (49.4%)
Technical Success	81 (100%)

Values are n (%) unless otherwise specified. IQR, interquartile range

112 Surpass Elite devices were deployed across the entire cohort with most treated with one device (75.3%, *n* = 61), 21.0% (*n* = 17) with 2 and 3.7% (*n* = 3) with 3 or more. Of the 81 included cases, upper extremity access (radial/ulnar) was performed in 55.6% (*n* = 45), transfemoral access in 42.0% (*n* = 34), and access via both in 2.5% (*n* = 2).

The median fluoroscopy time was 31.8 min (IQR: 25.5–40.8). Device diameters ranged from 3.25–5.25 mm, the most common diameters being 4.5, 5.0, and 4.0 mm. Device lengths ranged from 15–50 mm, with the most common lengths being 20, 25, 15, and 30 mm. Adjunctive balloon angioplasty and coil embolization was required in 13.6% (*n* = 11) and 9.9% (*n* = 8) of cases, respectively. Postoperative Dyna CT imaging was performed in 49.4% (*n* = 40).

### Angiographic outcomes

Angiographic follow-up was available for 60 cases (74.1%) with 61 aneurysms having complete RROC and OKM scoring at a mean follow-up of approximately 6 months **(**Table 4**)**. One case involved two aneurysms treated simultaneously, each with separate occlusion scores: the hypophyseal aneurysm achieved RROC Class I/OKM Grade D, while the ophthalmic aneurysm showed RROC Class IIIa/OKM Grade B. Follow-up imaging included DSA in 75.4% (*n* = 46), CTA in 19.7% (*n* = 12), and MRA in 4.9% (*n* = 3) of cases.

**Table 4:** Clinical and angiographic outcomes

Variable	Total
Angiographic Follow-up	
Cases with imaging follow-up	60/81 (74.1%)
Aneurysms with RROC/OKM scores*	61
Follow-up imaging modality (*n* = 61)	
DSA	46 (75.4%)
CTA	12 (19.7%)
MRA	3 (4.9%)
RROC Scale (*n* = 61 aneurysms)	
Class I (complete occlusion)	48 (78.7%)
Class II (residual neck)	0 (0%)
Class IIIa (residual aneurysm)	13 (21.3%)
Class IIIb (no change)	0 (0%)
OKM Scale (*n* = 61 aneurysms)	
Grade D (total occlusion)	48 (78.7%)
Grade C (subtotal/entry remnant)	2 (3.3%)
Grade B (neck remnant)	11 (18.0%)
Grade A (aneurysm filling)	0 (0%)
Adequate occlusion rate†	50/61 (82.0%)
Device-Related Findings at Follow-up	
In-stent stenosis (< 50%)	2/60 (3.3%)
ACA A1 origin stenosis (jailing)	2/60 (3.3%)
Clinical Outcomes	
mRS at follow-up (*n* = 59 cases)	
mRS 0	45 (76.3%)
mRS 1	12 (20.3%)
mRS 2	1 (1.7%)
mRS ≥ 3	1 (1.7%)
Good clinical outcome (mRS 0–2)	58/59 (98.3%)
Safety Outcomes	
Procedural complications	4 (4.9%)
Mortality	6 (7.4%)
Procedure-related	1 (1.2%)
Unrelated to procedure	5 (6.2%)
Discharge Timing	
POD 1	59 (72.8%)
POD 2	5 (6.2%)
POD ≥ 3	14 (17.3%)
Unknown	3 (3.7%)

^*^One case involved two aneurysms treated simultaneously with separate scores

^†^Adequate occlusion = RROC Class I–II or OKM Grade C–D

**Fig. 3:**
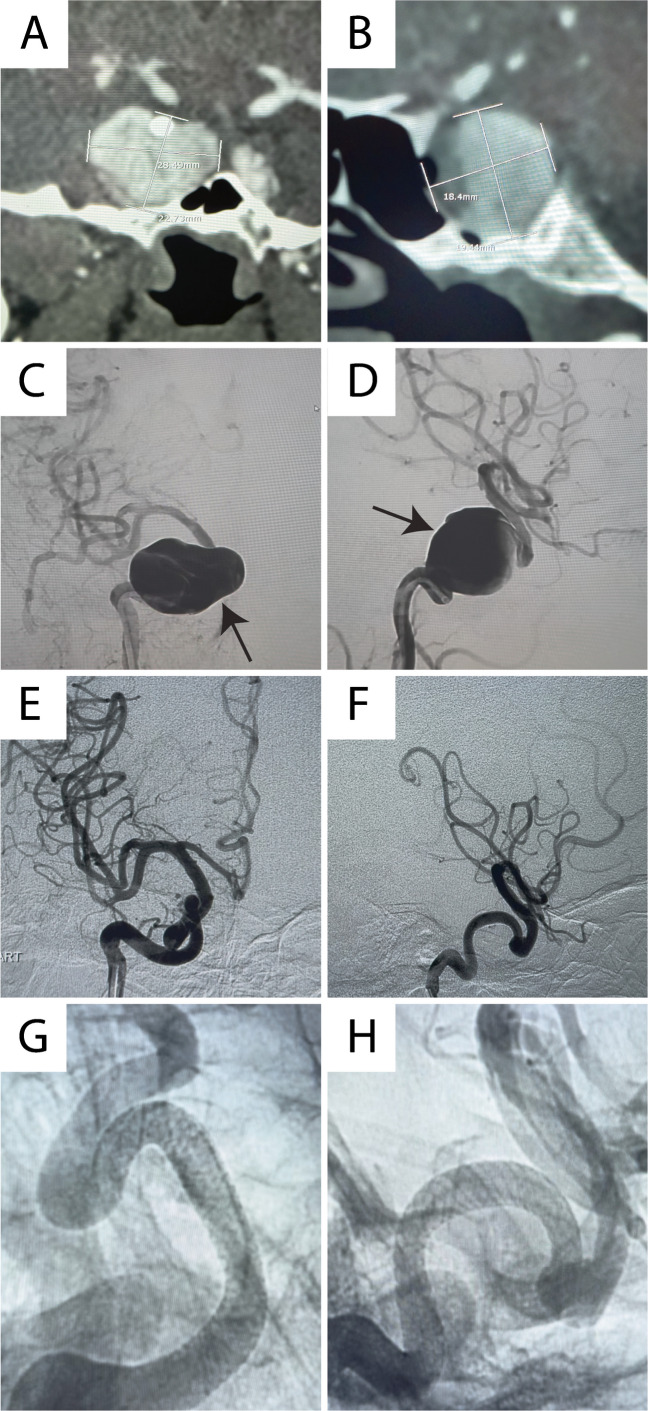
54-year-old Hispanic female with giant right internal carotid artery (ICA) cavernous aneurysm presenting with interval aneurysm growth and development of right sixth nerve palsy. **A**, **B** Coronal and sagittal computed tomography angiography (CTA) reconstruction views demonstrating a 22.8 × 26.8 mm (AP projection) and 24.8 × 21.3 mm (lateral projection) partially thrombosed giant right ICA cavernous aneurysm remodeling the sella turcica with expansion and bony erosion. **C**, **D** Pretreatment anteroposterior (AP) and lateral angiographic views of right ICA demonstrating the giant right ICA aneurysm (black arrow). **E**, **F** Six-month follow-up post-treatment AP and lateral angiographic views of right ICA demonstrating complete aneurysm obliteration with remodeling of the right ICA. Notably, there is improved filling of the intracranial circulation due to resolution of flow steal into the giant aneurysm. **G**, **H** Magnified oblique unsubtracted AP and lateral views of right ICA demonstrating the braid of the single Surpass Elite device (4.0 × 30 mm) and smooth remodeling of the cavernous right ICA with no residual aneurysm filling

OKM scoring demonstrated Grade D (total occlusion) in 78.7% (*n* = 48), Grade C (subtotal with entry remnant) in 3.3% (*n* = 2), Grade B (neck remnant) in 18.0% (*n* = 11), and Grade A (aneurysm filling) in 0% (*n* = 0). Complete occlusion (RROC Class I or OKM Grade D) was achieved in 78.7% (48/61), and adequate occlusion (RROC Class I-II or OKM Grade C-D) in 82.0% (50/61). Representative cases demonstrated favorable anatomic outcomes with smooth vessel remodeling and complete aneurysm obliteration **(**Figs. 2 and 3**)**.

**Fig. 4:**
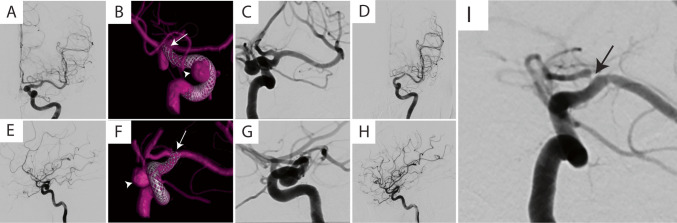
73-year-old Black female with unruptured left internal carotid artery (ICA) superior hypophyseal artery (SHA) aneurysm who presented with intermittent dizziness suggestive of vertigo. **A**, **B** Anteroposterior (AP) and lateral angiographic views of left ICA demonstrating a large left SHA aneurysm measuring 5.6 × 5.2 × 6.7 mm with a 4.5 mm wide base (black arrows). **C**, **D** AP and lateral oblique angiographic working projection views of left ICA demonstrating the aneurysm. **E**, **F** Dual-volume rotational 3D angiogram reconstructed images immediately post-treatment demonstrating the SHA aneurysm (white arrowhead) treated with two overlapping Surpass Elite devices (4.25 × 25 mm and 4.25 × 20 mm) with excellent visibility of the platinum braid. The construct extends from the left ICA terminus (white arrow) to the horizontal segment of left cavernous ICA, with the left anterior cerebral artery (ACA) origin jailed as a result. **G**, **H** Six-month follow-up AP and lateral angiographic views of left ICA demonstrating complete obliteration of the aneurysm with delayed filling of left ACA circulation. **I** Magnified oblique AP projection angiogram of left ICA at 6-month follow-up showing stenosis at the origin of the jailed left ACA (black arrow) secondary to flow diversion effect and competitive flow from the dominant right ACA

Mild in-stent stenosis (< 50%) was observed in 3.3% (2/60) of cases with follow-up imaging, and ACA A1 origin stenosis secondary to FD jailing effect in 3.3% (2/60) (Fig. 4).

### Clinical outcomes

Functional outcome data was available for 59 cases at follow-up **(**Table 4**)**. Of these, 98.3% (*n* = 58) achieved good functional outcome (mRS 0–2) while 1.7% (*n* = 1) did not (mRS ≥ 3).

Antiplatelet regimens at last follow-up (*n* = 61) included aspirin monotherapy in 42.6% (*n* = 26) and aspirin plus a P2Y12 inhibitor or anticoagulant in 57.4% (*n* = 35). Most patients were discharged on postoperative day 1 (72.8%, *n* = 59), with additional discharges on postoperative day 2 (6.2%, *n* = 5) and postoperative day ≥ 3 (17.3%, *n* = 14). Discharge timing was unknown in 3.7% (*n* = 3).

### Safety outcomes

Immediate intraprocedural complication rate was 2.5% (2/81), while the overall 30-day complication rate was 4.9% (4/81). Individual complications (Table 5A) included: Immediate intraprocedural complications (2/81, 2.5%): (1) microperforation with SAH and vasospasm (complete recovery, radial access, 2 devices, 2.9 mm aneurysm); and (2) intraoperative thrombosis managed with Integrilin and conversion to Brilinta (femoral access, 1 device, 4.6 mm aneurysm)—this patient was discharged without neurological deficit but returned the following day with an MCA territory stroke due to medication noncompliance with ticagrelor, resulting in significant morbidity. Delayed complications within 30 days (2/81, 2.5%): (3) stroke secondary to medication noncompliance (death, radial access, 2 devices, 3.0 mm aneurysm); and (4) groin hematoma (patient recovered, femoral access, 1 device, 7.3 mm aneurysm). Thus, two patients suffered strokes from antiplatelet medication noncompliance, underscoring the importance of adherence to antiplatelet regimens.

**Table 5:** Safety analysis

**A. Individual complication details (classified by timing)**				
**Complication**	**Access**	**Devices**	**Aneurysm Size (mm)**	**Outcome**
Microperforation with SAH/vasospasm	Radial	2	2.9	Recovered
Stroke (medication noncompliance)	Radial	2	3.0	Death
Groin hematoma	Femoral	1	7.3	Recovered
Intraoperative thrombosis*	Femoral	1	4.6	Delayed stroke†
Delayed right temporal hemorrhage‡	Radial	2	3.2	Managed medically
**B. Mortality analysis (*n* = 6)**				
**Cause of Death**	**Time to Death**	**Procedure-Related**	**Notes**	
Stroke from medication noncompliance	—	Yes	Patient noncompliance	
Large infarcts from post-SAH vasospasm	11 days	No	Ruptured aneurysm with SAH	
Recurrent left hemispheric ICH	3 weeks	No	Unrelated	
Acute respiratory failure/acute on chronic heart failure	5 months	No	Unrelated to device/procedure	
Homicide (stabbing)	~ 6 months	No	Unrelated	
Unknown	—	No	Cannot be determined	

30-day mortality: 2/81 (2.5%). Mortality beyond 30 days: 4/81 (4.9%). Overall mortality: 6/81 (7.4%). Procedure-related mortality: 1/81 (1.2%). Note: The post-SAH vasospasm death at 11 days may be attributable to the natural history of SAH rather than the device or procedure itself. The acute respiratory failure/acute on chronic heart failure death at 5 months was unrelated to the device or procedure

*Intraprocedurally resolved with Integrilin administration and conversion to ticagrelor; discharged without deficit. †Patient returned the following day with MCA territory stroke due to medication noncompliance with ticagrelor. Immediate intraprocedural complications: microperforation, intraoperative thrombosis. Delayed complications (within 30 days): stroke from noncompliance, groin hematoma. ‡Delayed adverse event beyond 30 days (*n* = 1): delayed right temporal intraparenchymal hemorrhage at 10 months in a patient on dual antiplatelet therapy plus apixaban, attributed to cerebral amyloid angiopathy; not considered device- or procedure-related

Mortality was 7.4% (6/81), 30-day mortality was 2.5% (2/81) and mortality beyond 30 days was 4.9% (4/81) (Table 5B). Deaths within 30 days included: (1) stroke from medication noncompliance (procedure-related); and (2) post-SAH vasospasm with large infarcts 11 days after treating a ruptured aneurysm (this death may be attributable to the natural history of SAH rather than the device or procedure itself, though a definitive distinction is difficult). Deaths beyond 30 days included: (3) recurrent hemispheric intracerebral hemorrhage at 3 weeks post-procedure (likely unrelated); (4) acute respiratory failure with acute on chronic heart failure at 5 months (unrelated to device or procedure); (5) homicide at approximately 6 months (unrelated); and (6) death of unknown cause (relationship to procedure cannot be determined). One delayed adverse event beyond 30 days was observed during extended follow-up: a delayed right temporal intraparenchymal hemorrhage at 10 months in a patient on dual antiplatelet therapy plus apixaban, attributed to cerebral amyloid angiopathy and not considered device- or procedure-related.

### Subgroup analysis

Comprehensive subgroup analyses were performed to evaluate the influence of patient and procedural characteristics on occlusion and functional outcomes **(**Table 6**).** Technical success was 100% across all subgroups. No significant differences in complete occlusion rates were observed by access site (transradial 84.8% vs transfemoral 72.0%; *p* = 0.33), circulation (anterior 80.8% vs posterior 66.7%; *p* = 0.39), rupture status (unruptured 80.8% vs ruptured 100%; *p* = 1.00), number of devices (1 device 74.4% vs ≥ 2 devices 91.7%; *p* = 0.26), age (< 60 years 80.0% vs ≥ 60 years 82.8%; *p* = 1.00), or sex (female 81.6% vs male 66.7%; *p* = 0.67). Good functional outcome (mRS 0–2) rates were similarly comparable across subgroups, ranging from 90.0% to 100%.

**Table 6:** Subgroup analysis of outcomes

Subgroup	n	Technical Success	Complete Occlusion*	Good Outcome†	*p*-value‡
Access Site					
Transradial	43	43 (100%)	28/33 (84.8%)	30/30 (100%)	0.33/1.00
Transfemoral	34	34 (100%)	18/25 (72.0%)	24/25 (96.0%)	
Circulation					
Anterior	67	67 (100%)	42/52 (80.8%)	49/50 (98.0%)	0.39/0.51
Posterior	14	14 (100%)	6/9 (66.7%)	8/8 (100%)	
Anatomy					
ICA: Carotid siphon	46	46 (100%)	26/31 (83.9%)	30/30 (100%)	—
ICA: Non-siphon	17	17 (100%)	12/14 (85.7%)	13/14 (92.9%)	
Posterior circulation	14	14 (100%)	6/9 (66.7%)	8/8 (100%)	
Other (MCA/ACA)	4	4 (100%)	1/3 (33.3%)	3/3 (100%)	
Aneurysm Size (< 7 mm cutoff)					
< 7 mm	64	64 (100%)	38/46 (82.6%)	36/38 (94.7%)	0.28/1.00
≥ 7 mm	17	17 (100%)	10/15 (66.7%)	16/16 (100%)	
Aneurysm Size (< 5 mm cutoff)					
< 5 mm	46	46 (100%)	29/31 (93.5%)	24/26 (92.3%)	0.005/0.08
≥ 5 mm	35	35 (100%)	19/30 (63.3%)	28/28 (100%)	
Aneurysm Size (by category)					
< 3 mm	13	13 (100%)	6/6 (100%)	5/5 (100%)	—
3–5 mm	33	33 (100%)	23/25 (92.0%)	19/21 (90.5%)	
5–7 mm	18	18 (100%)	9/15 (60.0%)	12/12 (100%)	
7–12 mm	13	13 (100%)	8/11 (72.7%)	12/12 (100%)	
13–25 mm	1	1 (100%)	1/1 (100%)	1/1 (100%)	
> 25 mm (Giant)	3	3 (100%)	1/3 (33.3%)	3/3 (100%)	
Rupture Status					
Unruptured	77	77 (100%)	42/52 (80.8%)	50/52 (96.2%)	1.00/1.00
Ruptured	4	4 (100%)	2/2 (100%)	2/2 (100%)	
Prior Treatment					
De novo	71	71 (100%)	44/53 (83.0%)	45/47 (95.7%)	0.09/1.00
Retreatment	9	9 (100%)	4/7 (57.1%)	6/6 (100%)	
Number of Devices					
1 device	61	61 (100%)	32/43 (74.4%)	40/42 (95.2%)	0.26/1.00
≥ 2 devices	20	20 (100%)	11/12 (91.7%)	12/12 (100%)	
Number of Devices × Size					
< 5 mm, 1 device	—	—	18/20 (90.0%)	—	—
< 5 mm, ≥ 2 devices	—	—	8/8 (100%)	—	
≥ 5 mm, 1 device	—	—	15/22 (68.2%)	—	
≥ 5 mm, ≥ 2 devices	—	—	3/4 (75.0%)	—	
Age					
< 60 years	36	36 (100%)	20/25 (80.0%)	25/25 (100%)	1.00/0.49
≥ 60 years	45	45 (100%)	24/29 (82.8%)	27/29 (93.1%)	
Sex					
Female	63	63 (100%)	40/49 (81.6%)	41/42 (97.6%)	0.67/0.40
Male	18	18 (100%)	8/12 (66.7%)	11/12 (91.7%)	

*Complete occlusion = RROC Class I. †Good outcome = mRS 0–2. ‡*P*-values: complete occlusion/good outcome (Fisher’s exact test). Bold *p*-value indicates statistical significance (*p* < 0.05). The “Number of Devices × Size” and “Aneurysm Size (by category)” rows report occlusion only; *p*-values are not calculated for individual strata due to small sample sizes. The “Anatomy” rows are descriptive; *p*-values are not calculated due to multiple small subgroups

Aneurysm size was the only characteristic reaching statistical significance as a predictor of occlusion. At the < 7 mm cutoff, complete occlusion was numerically but not significantly higher in smaller aneurysms (82.6% [38/46] vs 66.7% [10/15]; *p* = 0.28). However, a more discriminating cutoff at 5 mm revealed a statistically significant difference: aneurysms < 5 mm achieved 93.5% (29/31) complete occlusion compared with 63.3% (19/30) for aneurysms ≥ 5 mm (*p* = 0.005). Within individual size strata, complete occlusion rates were 100% (6/6) for < 3 mm, 92.0% (23/25) for 3–5 mm, 60.0% (9/15) for 5–7 mm, 72.7% (8/11) for 7–12 mm, 100% (1/1) for 13–25 mm, and 33.3% (1/3) for giant aneurysms. Previously treated aneurysms also showed numerically lower complete occlusion compared with de novo aneurysms (57.1% [4/7] vs 83.0% [44/53]; *p* = 0.09), though this did not reach statistical significance.

To assess whether multi-device usage confounded the reported occlusion rates, a stratified analysis by device number and aneurysm size was performed. Single-device treatment of aneurysms < 5 mm achieved 90.0% (18/20) complete occlusion, confirming robust single-device performance in this size range. Multi-device constructs showed numerically higher occlusion compared with single-device cases within each size stratum: for aneurysms < 5 mm, 100% (8/8) with ≥ 2 devices vs 90.0% (18/20) with 1 device; for aneurysms ≥ 5 mm, 75.0% (3/4) with ≥ 2 devices vs 68.2% (15/22) with 1 device. The numerically higher occlusion in the multi-device group likely reflects both increased metal coverage and anatomically-driven case selection, as multi-device constructs were preferentially employed in carotid siphon locations (55% of multi-device cases), posterior circulation aneurysms (25%), fusiform morphology (20%), large or giant aneurysms (25%), and cases with multiple ipsilateral aneurysms (20%).

## Discussion

This multicenter retrospective study is the first reported clinical experience with the Surpass Elite FD, demonstrating 100% technical success, 78.7% complete occlusion, and 98.3% good functional outcomes (mRS 0–2) at 6-month follow-up, providing preliminary evidence for this third-generation device.

### Technical performance and device deliverability

The 100% technical success rate observed in our series is consistent with published data for the PED, which was successfully deployed in 93.9–99.3% of patients in large registries [3, 26–29]. Likewise, the Surpass Streamline achieved 97.8% technical success in the SCENT trial while the Surpass Evolve was successfully implanted in all patients per a meta-analysis of 8 studies [10, 11]. Our experience suggests that Surpass Elite maintains the reliable deliverability established by its predecessors while incorporating improvements.

The low rate of adjunctive balloon angioplasty (13.6%) in our series contrasts historical experience using the Surpass Evolve, which mandated balloon angioplasty in 26.1% of cases [11]. This may in part reflect the increased braid angle (> 150 degrees) to enhance spontaneous device opening and wall apposition. If confirmed in larger studies, this reduction could translate to shorter procedures, decreased radiation, and potentially reduced risk of vessel injury or device disruption; however, operator experience and case selection may also contribute to the observed difference [30, 31].

Furthermore, lack of braid deformations or device migration events at 6-month follow-up is reassuring and suggests that the design modifications do not compromise mechanical stability. This finding is important given that braid deformation has been reported as a rare significant FD complication [32].

### Angiographic & clinical outcomes

Complete occlusion was observed in 78.7% of aneurysms and adequate occlusion in 82.0% at 6-month follow-up. These rates are within the range reported for established FDs, though direct comparison is limited by differences in study design, patient selection, and follow-up duration. The Surpass Streamline reported 62.8% complete occlusion at 12 months, while a meta-analysis of Surpass Evolve studies showed 67.1% occlusion rates at 8 months [10, 31]. The PED demonstrated 69.2–75% complete occlusion at 6 months across five cohorts, and the FRED FD achieved 73% at 6 months [33–35]. Direct comparison across devices remains challenging due to differences in follow-up timepoints, patient selection, and study design. However, an in vitro study highlighted that Surpass Elite’s higher pore density (18–25/mm^2^) and prolonged turnover time (1.2–25.2%) compared to PED-Shield may enhance flow reduction and aneurysmal stagnation [36].

The size distribution of our cohort warrants consideration when interpreting occlusion rates. Aneurysms < 5 mm achieved significantly higher complete occlusion than those ≥ 5 mm (*p* = 0.005), confirming that the high proportion of small aneurysms favorably influenced the overall rate (Table 6), consistent with the Surpass Evolve experience by Gupta et al. demonstrating that aneurysms < 10 mm achieve significantly higher 6-month occlusion rates [37]. While direct comparison with early Pipeline registries consisting of predominantly large and giant aneurysms is therefore not appropriate given the fundamentally different case mix, the PREMIER trial validated flow diversion for aneurysms ≤ 12 mm [38], and Chalouhi et al. demonstrated comparable safety and a trend toward higher occlusion with PED compared to stent-assisted coiling for small unruptured aneurysms [39]. In a separate matched comparison, the same group showed significantly higher occlusion with flow diversion than coiling alone in small, noncomplex aneurysms [40]. Treatment decisions for small aneurysms in our cohort were informed by patient-specific risk factors including young age, documented growth, family history, and clinical presentation, reflecting the ongoing expansion of flow diversion indications.

Beyond aneurysm size, several cohort characteristics merit attention. Hypertension was present in 61.5% of patients and 85.2% of aneurysms were saccular—both factors associated with lower PED occlusion rates [41]. Additionally, 21% of aneurysms were > 7 mm and 11.1% were previously treated, characteristics linked to reduced occlusion in prior literature [42, 43]. Subgroup analysis showed numerically lower occlusion in both groups, with prior treatment approaching significance (*p* = 0.09; Table 6), though the study was underpowered for these comparisons.

Functional outcomes were favorable, with 98.3% of patients achieving mRS 0–2 at follow-up (76.3% mRS 0, 20.3% mRS 1). These results align with published FD series wherein 100% of patients experienced good functional outcome with the FRED X at 6 months, while the Surpass Streamline and PED achieved 82.2% improved/stable and 97% good functional outcome rates at 1-year follow-ups, respectively [10, 44, 45]. The 72.8% discharge rate on postoperative day 1 reflects contemporary perioperative protocols and suggests the Surpass Elite is suitable for ambulatory or short-stay settings, with implications for healthcare efficiency and patient satisfaction.

### Safety profile

The immediate intraprocedural complication rate of 2.5% and the overall 30-day complication rate of 4.9% in our series are lower than the 13.5% and 8% complication rates reported by meta-analyses of PED and Surpass Evolve studies, respectively, although differences in study design and reporting preclude direct comparison [11, 34]. Likewise, the SCENT trial for the Surpass Streamline reported an 8.3% rate of major adverse events within 12 months [10]. Our procedure-related mortality rate of 1.2% is consistent with the 2.1–3.3% rate observed in PED literature [33, 34].

Notably, the low rate of in-stent stenosis (3.3%) and minimal intimal hyperplasia observed at 6-month follow-up is encouraging and may represent an advantage of the Surpass Elite. Published literature reports in-stent stenosis rates of 4–15% for Pipeline and 8–12% for Surpass Evolve at similar timepoints. The consistently low stenosis rates observed may be related to the BioStealth surface charge neutralization technology, which is designed to reduce platelet activation and potentially limit the neointimal response. The total stenotic complication rate (mild in-stent stenosis 3.3% and ACA jailing 3.3%, totaling 6.6%) is consistent with or lower than stenosis-related complication rates reported in PED literature [34].

**Fig. 5:**
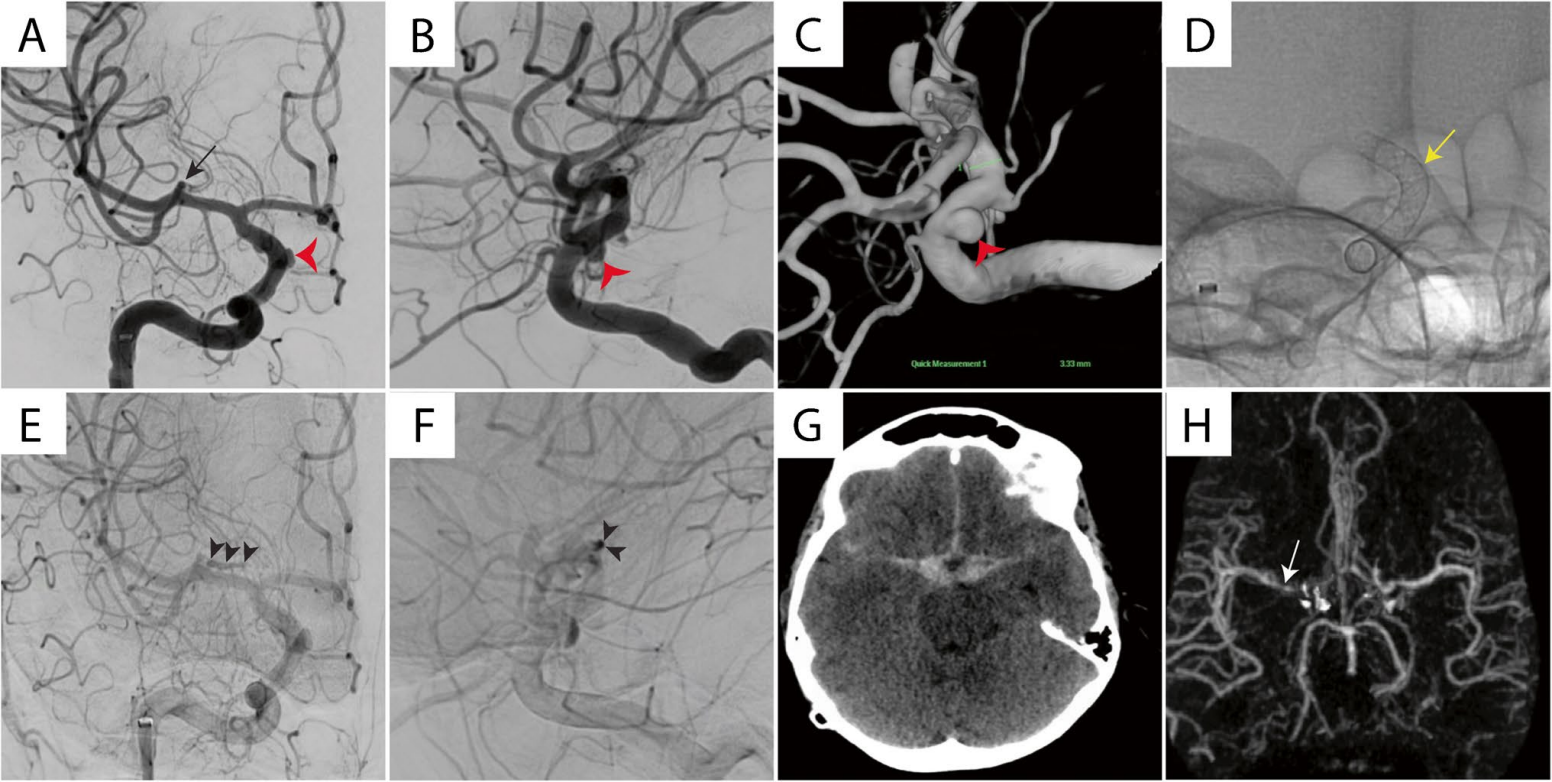
47-year-old female with unruptured right internal carotid artery (ICA) paraclinoid aneurysm treated with Surpass Elite flow diverter complicated by microperforation and subarachnoid hemorrhage. **A**, **B** Anteroposterior (AP) and lateral oblique angiographic views of right ICA injection demonstrating a right superior hypophyseal artery aneurysm measuring 2.3 × 2.7 × 2.9 mm with a 3.0 mm neck (red arrowhead). **C** Rotational 3D reconstruction demonstrating the right ICA aneurysm (red arrowhead). **D** Magnified unsubtracted radiographic view with Surpass Elite flow diverter deployed, showing good device visualization (yellow arrow). **E**, **F** AP and lateral oblique angiographic views of right ICA injection post-Surpass deployment in the early parenchymal phase demonstrating extravascular slow contrast stasis indicative of microperforation around a dominant lateral lenticulostriate artery, which was managed with balloon inflation, heparin reversal and subsequent complete resolution. **G** Postoperative axial non-contrast head computed tomography demonstrating subarachnoid hemorrhage in the basal cisterns. **H** Computed tomography angiography reconstruction on postoperative day 7 demonstrating moderate vasospasm in the right M1 segment. The patient was premedicated with dual antiplatelet therapy consisting of aspirin and Brilinta, with P2Y12 reaction units measured at 5, indicating hyperresponder status with excellent platelet inhibition

Among procedure-related complications in our series, only 1.2% (1/81) were device-related: a microperforation with SAH and vasospasm that was successfully managed with complete recovery (Fig. 5). This is lower than the 3.5% intraprocedural complication rates experienced using PED [34]. A meta-analysis of PED studies involving 1915 patients also reported an additional 4.7% ischemic events and 2.7% intracranial hemorrhages [34]. Additionally, an in vitro comparison of the Surpass Elite to the PED-Shield highlighted Elite’s significantly reduced inflow rate and impact zone, which may decrease the risk of aneurysm rupture [36]. This aligns with our study’s findings, in which the 21% of aneurysms > 7 mm in our cohort were successfully treated without device-related complications despite being at higher risk of aneurysm rupture [46].

Delayed complications within 30 days (2.5%) were attributable to antiplatelet non-compliance (stroke with fatal outcome) and access site issues (groin hematoma with full recovery). Importantly, two patients in this series suffered strokes due to antiplatelet medication noncompliance: one fatal and one resulting in significant morbidity. This pattern suggests that the device itself performed safely, with only one device-related complication that resolved completely, while other complications were primarily associated with patient-specific factors, particularly antiplatelet adherence, which remains a critical determinant of outcomes following FD.

### Technical considerations and practical insights

Our experience provides several practical insights for clinicians adopting the Surpass Elite. First, the device performed reliably across both transradial (53.1%) and transfemoral (42.0%) routes, with no significant differences in outcomes, supporting compatibility with the increasing adoption of transradial access [47, 48].

Second, the device showed versatility in treating diverse aneurysm locations and configurations. While 75.3% of aneurysms were in the ICA (reflecting typical FD indications), successful treatment was also observed in aneurysms of the posterior circulation (17.3%) and those at other locations, consistent with present literature on the Surpass Evolve [3, 11]. Furthermore, successful treatment across a range of aneurysm diameters (16.0% < 3 mm, 40.7% 3–5 mm, 22.2% 5–7 mm, 16.0% 7–12 mm, 1.2% 13–25 mm, and 3.7% giant > 25 mm) is relevant considering ongoing expansion of flow diversion indications to include high risk smaller-midsized lesions [27].

**Fig. 6:**
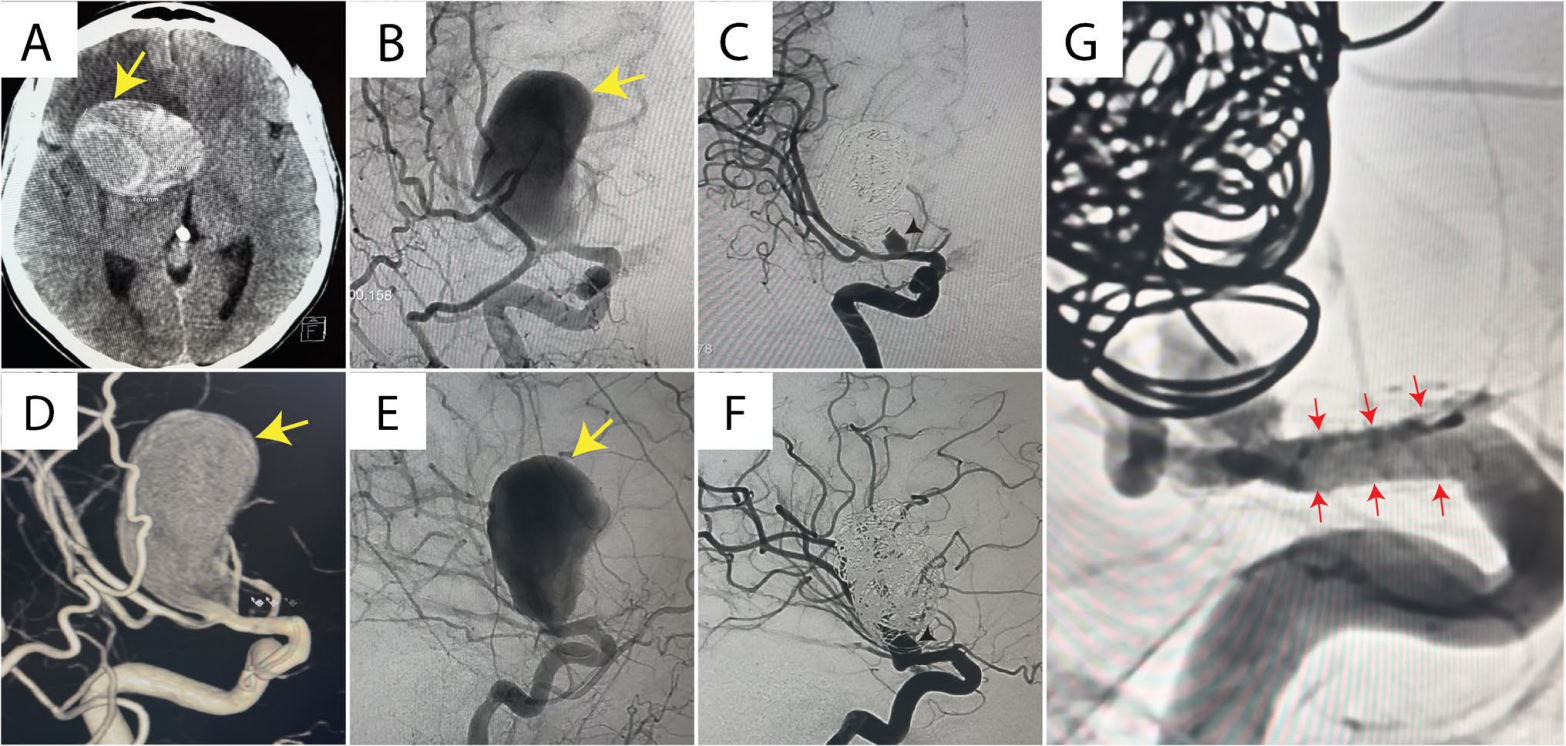
51-year-old Caucasian female with unruptured giant partially thrombosed right middle cerebral artery (MCA) aneurysm causing mass effect and presenting with syncope, treated with coil embolization and Surpass Elite flow diverter. **A** Axial non-contrast head computed tomography demonstrating partially thrombosed giant aneurysm causing mass effect (yellow arrow). **B**, **E** Anteroposterior (AP) and lateral angiographic views of right internal carotid artery (ICA) demonstrating filling of a giant proximal right M1 MCA aneurysm measuring 35 × 21 × 31 mm with a 5.6 mm neck (yellow arrow) with irregular contours and superior projection. **D** Rotational 3D angiogram demonstrating filling of the giant right MCA aneurysm. **C**, **F** Three-month follow-up AP and lateral angiographic views of right ICA after placement of coils and single Surpass Elite flow diverter (3.75 × 15 mm) extending from the ophthalmic segment of right ICA to the mid-M1 segment across the aneurysm neck, demonstrating complete dome obliteration with residual filling of the aneurysm neck (black arrowhead). **G** Magnified unsubtracted lateral projection angiogram of right ICA showing residual aneurysm neck and remodeling of the parent ICA with minimal intimal hyperplasia (red arrows)

Third, the relatively low rate of adjunctive coiling (9.9%) suggests the device can effectively treat most aneurysms with flow diversion alone, though strategic use of coiling remains important for specific scenarios such as large or giant aneurysms where immediate thrombosis may be desirable to prevent continued expansion or rupture (Fig. 6).

Fourth, the biopassive surface treatment is a novel feature designed to reduce thrombogenicity through charge neutralization. While our study did not evaluate the impact of this surface modification, only one case of intraoperative thrombosis was identified and managed acutely with Integrilin and conversion to ticagrelor; this patient was discharged without deficit but subsequently suffered a stroke due to medication noncompliance the following day, emphasizing that successful intraprocedural management must be followed by strict adherence to antiplatelet therapy [49, 50]. The theoretical advantages of reduced platelet activation could have implications for future antiplatelet management strategies, potentially permitting de-escalation of DAPT in selected patients. However, such de-escalation must be approached cautiously and individualized based on patient-specific factors. This is considering that one patient in our series developed stroke owing to non-compliance to antiplatelet therapy, thus underscoring the critical importance of maintaining adequate platelet inhibition.

Next, multiple overlapping devices were employed in 24.7% of cases, predominantly at carotid siphon locations (55% of multi-device cases), with additional indications including posterior circulation anatomy, fusiform morphology, large aneurysms with wide necks, and multiple ipsilateral aneurysms. The decision to deploy additional devices was driven by the need to achieve adequate metal coverage across the aneurysm neck, particularly in the geometrically complex carotid siphon where vessel curvature reduces effective device coverage at the outer curve. Shapiro et al. demonstrated that even modest oversizing of a single flow diverter leads to a significant increase in porosity, and that metal coverage is reduced at the outer curvature of deployed devices [51]. In a companion study, the same group showed that building multidevice constructs using devices of different diameters can achieve more uniform coverage patterns, with overlapping two appropriately sized devices reliably providing coverage of 30% and higher—values rarely attainable with single-device use [52]. Among the four complications in our series, two occurred in multi-device cases and two in single-device cases, with only one complication across all cases being device-related (microperforation, complete recovery). This finding is consistent with Vranic et al., who reported no significant difference in occlusion or complication rates between single and multiple PED constructs in a multicenter study of 298 patients [53].

Lastly, the perioperative use of corticosteroids in large and giant aneurysm cases was informed by evidence that flow diversion–induced thrombosis can provoke perianeurysmal brain inflammation, manifesting as transient worsening of headaches and compressive symptoms within days of treatment. Berge et al. demonstrated this inflammatory response on MRI in 41% of flow diverter–treated patients, with larger aneurysm size predictive of its occurrence, and proposed that thrombosis-mediated endothelial cell death triggers release of damage-associated molecular patterns, inflammasome activation, and IL-1β production via NF-κB signaling — a cascade that corticosteroids may attenuate through inhibition of NF-κB translocation [23]. While steroid efficacy has not been established in a controlled setting, and Berge et al. noted that steroids failed to prevent inflammation in the largest aneurysms in their series, similar perioperative steroid protocols have been adopted by other groups [24, 25]. The rationale for prophylactic corticosteroid use is further supported by the recognized risk of thrombus-associated mural autolysis in large and giant aneurysms, as described by Kulcsar et al. [54].

### Study limitations & future directions

Several limitations must be acknowledged. The retrospective design introduces potential selection bias. Additionally, with 74.1% angiographic and 72.8% clinical follow-up, our data may be subject to attrition bias if patients with adverse or suboptimal outcomes were differentially likely to return for follow-up. Our mean follow-up duration of approximately 6 months is relatively short for flow diversion, where progressive aneurysm occlusion can last longer [55]. The incomplete follow-up rate raises the possibility that patients with adverse or favorable outcomes were differentially lost, potentially biasing the reported occlusion and complication rates. Longer follow-up is essential to assess durability of occlusion and delayed complications such as late aneurysm rupture, progressive in-stent stenosis, and parent vessel narrowing.

The relatively small sample size (81 cases from 78 patients) limits statistical power for subgroup analyses, precluding definitive conclusions about outcome comparisons across patient and aneurysm characteristics. Additionally, 24.7% of cases (20/81) required multiple overlapping devices, which limits our ability to isolate single-device performance characteristics; outcomes in these cases may reflect the combined effect of multiple devices rather than individual device performance alone [53]. Notably, although procedures were performed across four centers, all were carried out by only two experienced operators, which limits the generalizability of these findings to the broader neurointerventional community and introduces potential operator-dependent bias. The term “multicenter” should therefore be interpreted in this context. Finally, the lack of a control group prevents direct comparison of the Surpass Elite to other FDs, and the study period (January-July 2025) captures early post-approval experience that may reflect an operator learning curve.

Despite these limitations, our findings establish a foundation for future research on the Surpass Elite. Prospective multicenter studies with long-term follow-up are needed to confirm and extend these initial observations. Comparative studies against other contemporary FDs, ideally in randomized or propensity-matched designs, would help optimize patient selection for each device.

## Conclusions

This study presents the first published clinical experience with the Surpass Elite flow diverter, demonstrating successful device implantation in all 81 cases, 78.7% complete aneurysm occlusion (RROC Class I/OKM Grade D), and 98.3% good functional outcomes (mRS 0–2) at a mean follow-up of approximately 6 months. The safety profile was satisfactory, with a 2.5% immediate intraprocedural complication rate, a 4.9% 30-day complication rate, and a 1.2% procedure-related mortality; mild in-stent stenosis was observed in only 3.3% of cases with follow-up imaging. The low rate of adjunctive balloon angioplasty (13.6%) may in part reflect the clinical benefit of the modified braid architecture, though operator experience and case selection cannot be excluded as contributing factors. These findings should be interpreted in the context of a retrospective design, incomplete and relatively short follow-up, and the involvement of only two operators, which limits the generalizability of these early observations. Prospective studies with longer follow-up and larger sample sizes, ideally with comparator flow diverters in randomized or propensity-matched designs, are needed to confirm these preliminary findings and define the role of the Surpass Elite among contemporary flow diverters.

## Supplementary Information

Below is the link to the electronic supplementary material.

Supplementary file1 (DOCX 20 KB)

## Data Availability

The data that support the findings of this study were generated from retrospective clinical records and imaging data from participating institutions. Due to patient privacy and institutional regulations, the data are not publicly available.
