# Supplementary material for: First clinical experience with the surpass elite flow diverter in the treatment of intracranial aneurysms
Source: Neuroradiology. 2026 May 2;68(6):1575–92. doi: 10.1007/s00234-026-04010-y (PMC13323425; doi:10.1007/s00234-026-04010-y)
Supplement: Supplementary file 1 — Supplementary file1 (DOCX 20 KB) [file 234_2026_4010_MOESM1_ESM.docx]

**SUPPLEMENTARY MATERIALS**

**Supplementary Table S1. Comparison of Contemporary Flow Diverter Device Specifications**

| **Feature** | **Surpass Elite** | **Surpass Evolve** | **Surpass Streamline** | **PED Shield** | **PED Vantage** | **FRED X** | **p64** | **p48 MW** |
| --- | --- | --- | --- | --- | --- | --- | --- | --- |
| **Manufacturer** | Stryker | Stryker | Stryker | Medtronic | Medtronic | MicroVention (Terumo) | phenox (Wallaby Medical) | phenox (Wallaby Medical) |
| **Wire count** | 64 | 64 | 72 (3–4mm); 96 (5mm) | 48 | 48 or 64 | 64 total (48 inner + 16 outer, dual-layer) | 64 | 48 |
| **Wire construction** | 52 pure CoCr + 12 PtW | 52 pure CoCr + 12 PtW | CoCr-Ni + PtW | 36 pure CoCr + 12 PtW | DTF (Pt core / CoCr sheath) | Nitinol (dual-layer); interwoven tantalum helical markers | Nitinol | DFT (Pt-filled nitinol) |
| **Braid angle** | 150–158° | 133–152° | NR | NR | NR | NR | NR | NR |
| **Diameters (mm)** | 3.25–5.25 (8 sizes) | 3.25, 4.0, 4.5, 5.0 | 3.0, 4.0, 5.0 | 2.5–5.0 (0.25mm steps) | 2.5–5.0 (0.25mm steps) | 2.5–5.5 (0.5mm steps) | 2.5–5.0 | 2.0, 3.0 |
| **Lengths (mm)** | 12–50 | 12–40 | 15–50 | 10–35 | 10–35 | 13–36 | NR | NR |
| **Indicated vessel (mm)** | 2.5–5.25 | 2.5–5.0 | 2.5–5.3 | 2.5–5.0 | 2.5–5.0 | 2.0–5.0 | 2.5–5.0 (p64 MW) | 1.75–3.0 |
| **Porosity range** | 68–79% | 64–79% | 67–75% | ~70% | ~70% | NR | NR | NR |
| **Mesh density (pores/mm²)** | 13.3–30.8 | 13.7–31.5 | 13.5–29.0 | NR | Higher than Flex (64-wire) | NR | NR | NR |
| **Metal coverage** | ~30% | ~30% | ~30% | ~30% | ~30% | 33–44% (dual-layer) | NR | NR |
| **Foreshortening** | 42–61% | 35.5–59% | 28–38% | 50–60% | 47–58% | NR | NR | NR |
| **Surface modification** | BioStealth (charge neutralization) | None | None | Shield (phosphorylcholine) | Shield (phosphorylcholine) | X Technology (poly-2-methoxyethyl acrylate) | None (HPC version available) | None (HPC version available) |
| **Heat treatment** | Multistage optimized | Single stage | NR | NR | NR | NR | NR | NR |
| **Delivery catheter** | XT-27 (0.027”) | 0.027” | Over-the-wire | Marksman (0.027”) | 0.021” or 0.027” | 0.021” (≤3.0mm) or 0.027” (≥3.5mm) | 0.027” (p64); 0.021” (p64 MW) | 0.021” (p48 MW); 0.017” (p48) |
| **Deployment mechanism** | Push/pull with resheathing | Push/pull with resheathing | Over-the-wire (not resheathable) | Push/pull with resheathing | Push/pull with resheathing | Push/pull with resheathing (up to 80%) | Mechanically detached; fully recoverable until detachment | Push/pull with resheathing (up to 80%) |
| **FDA status** | Cleared June 2024 | Cleared 2019 | Cleared 2018 | Approved April 2021 (PMA) | FDA approved; 027 model withdrawn Jan 2025 (Class I recall); 021 remains on market with updated IFU | FRED X approved Sept 2021 (PMA); original FRED approved Jan 2020 | Not FDA cleared | Not FDA cleared |
| **CE mark** | September 2024 | 2019 | 2016 | 2015 | CE marked | 2013 (FRED); FRED X CE marked | 2012 (p64); January 2020 (p64 MW) | CE marked (p48 MW) |

*CoCr = cobalt-chromium; PtW = platinum-tungsten; DTF = drawn filled tube; DFT = drawn filled tubing; PED = Pipeline Embolization Device; FRED = Flow Re-direction Endoluminal Device; HPC = hydrophilic polymer coating; IFU = instructions for use; NR = not reported.*

**Supplementary Table S2. Bench and Preclinical Performance Data for Flow Diverter Devices**

| **Parameter** | **Surpass Elite (BioStealth)** | **Surpass Evolve / Unmodified Elite** | **PED Shield** |
| --- | --- | --- | --- |
| **Surface modification mechanism** | Charge neutralization of outermost 4 nm; equalizes Cr/Co oxide (+) and Si/Mn oxide (–) surface compounds | None | 3 nm phosphorylcholine coating mimicking erythrocyte membrane |
| **GLP endothelialization (rabbit aorta, 30 days, n=18)** | Healthy endothelialized neointima | Healthy endothelialized neointima (comparator) | NR for this model |
| **SEM wire surface (2000×/5000×; new sterile units)** | Smooth, uniform | N/A | Surface irregularities visible |
| **Yield stress** | Higher than Evolve | Reference | NR |
| **Peak tracking force, largest available size (n=3)** | Lower than Evolve and PED Shield (at larger device size: 5.25×50mm) | Reference (5.0×40mm) | Higher than Evolve (5.0×35mm) |
| **Braid angle** | 150–158° | 133–152° | NR |
| **Mid-section opening (bench)** | Improved spontaneous opening vs. Evolve | Reference | N/A |
| **Ribboning behavior (bench)** | Reduced vs. Evolve | Reference | N/A |
| **Delivery wire** | Anatomy-specific stainless steel solid core; stiffness varies by arterial segment | Same platform | Guidewire-based; stainless steel core |
| **Resheath pad** | Higher durometer for improved proximal opening | Single durometer | Bumper with resheathing pad |

*TGA = thrombin generation assay; GLP = good laboratory practices; SEM = scanning electron microscopy; NR = not reported; N/A = not applicable.*
